# Comparative analysis of primary repair vs resection and anastomosis, with laparostomy, in management of typhoid intestinal perforation: results of a rural hospital in northwestern Benin

**DOI:** 10.1186/1471-230X-13-102

**Published:** 2013-06-19

**Authors:** Roberto Caronna, Alassan Kadiri Boukari, Dieudonnè Zaongo, Thierry Hessou, Rènè Castro Gayito, Cesar Ahononga, Sosten Adeniran, Giambattista Priuli

**Affiliations:** 1 Department of Surgical Sciences, Sapienza University of Rome, Policlinico Umberto I Viale del Policlinico 155, 00161 Rome, Italy; 2 Hôpital Saint Jean de Dieu, B.P. 7, Tanguièta, Benin

## Abstract

**Background:**

The objective is to compare primary repair vs intestinal resection in cases of intestinal typhoid perforations. In addition, we hypothesised the usefulness of laparostomy for the early diagnosis and treatment of complications.

**Methods:**

111 patients with acute peritonitis underwent emergency laparotomy: number of perforations, distance of perforations from the ileocaecal valve, and type of surgery performed were recorded. A laparostomy was then created and explored every 48 to 72 hours. The patients were then divided into two groups according to the surgical technique adopted at the initial laparotomy: primary repair (Group A) or intestinal resection with anastomosis (Group B). Clinical data, intraoperative findings, complications and mortality were evaluated and compared for each group.

**Results:**

In 104/111 patients we found intestinal perforations, multiple in 47.1% of patients. 75 had primary repair (Group A) and 26 had intestinal resection with anastomosis (Group B). Group B patients had more perforations than patients in Group A (p = 0.0001). At laparostomy revision, the incidence of anastomotic dehiscence was greater than that of primary repair dehiscence (p = 0.032). The incidence of new perforations was greater in Group B than in Group A (p = 0.01). Group B correlates with a higher morbility and with a higher number of laparostomy revisions than Group A (p = 0.005).

There was no statistical difference in terms of mortality between Group A and Group B. Presence of pus in the abdominal cavity at initial laparotomy correlates with significantly higher mortality (p = 0.0001).

**Conclusions:**

Resection and anastomosis shows greater morbidity than primary repair. Laparostomy revision makes it possible to rapidly identify new perforations and anastomotic or primary repair dehiscences; although this approach may seem aggressive, the number of operations was greater in patients who had a favourable outcome, and does not correlate with mortality.

## Background

Typhoid fever remains a notable public health issue in regions without adequate infrastructure [1,2]. It is generally transmitted by the faecal-oral route and is often endemic [1-4]. Asefa notes typhoid perforations as one of the most important causes underlying the acute abdomen in endemic regions [5]. Mortality rates of typhoid intestinal perforations are reported to be between 5% and 62% [6-8] but reach 80% in patients who receive late surgical treatment [9-14]. The mortality reported in developing countries is related to various factors, including sepsis (diffuse peritonitis), delayed treatment, malnutrition of many patients, age (many patients are young children), inadequate antibiotic therapy, and the scarcity or total absence of therapeutic resources [15-19]. Moreover, patients who receive surgical treatment may develop new perforations postoperatively, while anastomotic dehiscence, encountered in 5 to 15% of cases after intestinal resection, is considered a catastrophic complication [6,7,10,11,20].

Management of these patients is therefore complex, not only as regards choosing the most suitable surgical treatment (primary repair or intestinal resection) but also as regards an early diagnosis of complications (anastomotic or primary repair dehiscence, new perforations, endoabdominal abscesses), which can be difficult in the absence of diagnostic instruments, such as ultrasound and CT, that are often not available in rural hospitals in developing countries.

The purpose of our study was to analyse retrospectively, in a large hospital in Benin, the surgical treatment of patients with acute severe peritonitis and shock resulting from typhoid perforation. Surgical treatment of the perforations by primary repair is compared with intestinal resection with anastomosis, in order to provide guidelines for the safer choice of treatment. In addition, we evaluate the usefulness of a systematical adoption of laparostomy, which would allow re-exploration of the abdomen after 48 to 72 hours.

## Methods

111 patients with acute peritonitis and shock due to typhoid ileal perforation, who underwent surgery between January 2011 and January 2012 at the Hôpital Saint Jean de Dieu, Tanguièta, Benin, form the basis for this study. The hospital is a 300-bed facility serving a predominantly rural population. It has basic operative facilities, but supportive care such as postoperative mechanical ventilation, parenteral nutrition and invasive monitoring are unavailable. Because there were no pathologic or microbiologic services available, only patients who had typical operative findings of antimesenteric perforation of the distal ileum, consistent with typhoid fever, and severe general conditions, were included. Preoperative shock was defined as a preoperative systolic blood pressure of less than 90 mmHg.

Patient data were collected retrospectively from medical records and operating room registries. Age, sex, and laboratory tests, when available, were recorded. A plain abdominal radiography was made occasionally but it was not possible to collect retrospective data because the radiologist did not provide written reports, and the X-rays themselves remained in the possession of the patients.

All patients were very ill at presentation, requiring correction of fluid and electrolyte imbalance, and blood transfusion in some cases. Persistent abdominal pain of sudden onset with abdominal distension was regarded as clinical evidence of intestinal perforation.

Patients were resuscitated with intravenous fluids (glucose 5%, saline or lactated solution) either until an improvement, at least partial, of haemodynamic parameters (blood pressure, heart rate and diuresis) was obtained or, in any case, for no more than 3 hours. Nasogastric and vescical catheters were positioned in all cases.

In all cases, laparotomy was performed by inferior midline incision, prolonged upward if necessary, under general endotracheal anaesthesia with halothane. The number of perforations, the distance of the perforations from the ileocaecal valve, and the type of surgery performed (primary repair, intestinal resection with anastomosis, or other) were considered and recorded.

In cases of intestinal resection, a primary anastomosis was created using a continuous single-layer suture or, less frequently, a continuous double-layer suture, according to the surgeon’s experience.

**Figure 1:**
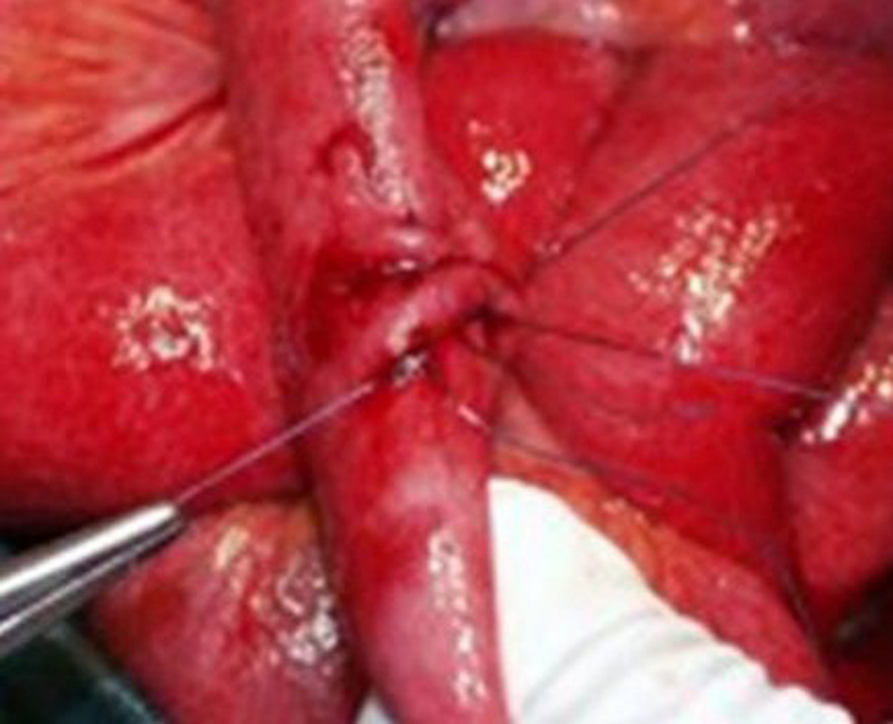
Primary repair technique.

Regarding primary repair a particular technique was always adopted, consisting of a single-layer suture with 2 to 4 large vicryl 2/0 “U” stitches passed through the seromuscular intestinal layer at some distance from the perforation, where there was less inflammatory involvement, achieving a good introflection of the perforation itself and without edge excision (Figure 1).

The peritoneal cavity was thoroughly cleaned with warm saline.

**Figure 2:**
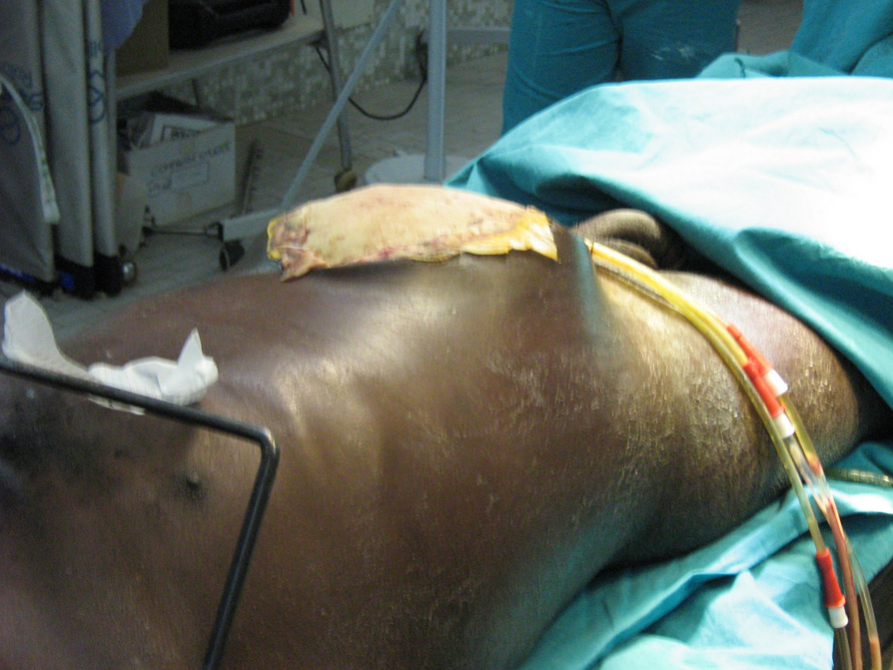
Laparostomy and abdominal drains.

For all patients, at the end of laparotomy, a laparostomy was created by fixing a grease gauze to the open aponeurosis with a continous non-absorbable suture. Three intraperitoneal drains were then placed: one in the right parietocolic gutter, one in the left parietocolic gutter, and one in the Douglas space (Figure 2). The laparostomy was then covered with 2 large laparotomic gauzes.

In the postoperative period, patients were not allowed to take food or fluid, and were given intravenous infusion of glucose and electrolyte solutions. The nasogastric catheter was maintained until faecal canalisation was achieved.

Antimicrobial combinations which included metronidazole with gentamycin and chloramphenicol were administered intravenously in the majority of patients. Other combinations used included metronidazole with either ceftriaxone or ciprofloxacin. Antibiotic therapy was administered until resolution of peritonitis and disappearance of fever.

The laparostomy was then explored, in the operating room, every 48 to 72 hours, until resolution of peritoneal contamination and complete consolidation of the sutures or the anastomosis. At each laparostomy revision, any primary repair or anastomotic dehiscences and appearance of any new perforation were recorded together with the relative treatment. If there were no signs of perforation or of pus in the peritoneum, the laparostomy was closed definitively, always leaving three intraperitoneal drainage catheters in the same positions; these were removed in the following days, according to the type and quantity of secretions. Closure of the fascial plane was always made with single stitches and never with continuous suture, because of the high risk of wound infection and dehiscence of laparotomy closures, as reported in literature [17].

All patients were observed until discharge. The patients were then divided into two groups according to the surgical technique adopted at the initial laparotomy: primary repair (Group A) or intestinal resection with anastomosis (Group B). Clinical data, intraoperative findings, complications and mortality for each group were evaluated and compared.

This retrospective analysis was approved by the Ethics Committee of the Hôpital Saint Jean de Dieu, Tanguièta, Benin.

### Statistical analysis

Statistical analysis was performed using the Student t-test or one-way ANOVA, as appropriate. A p-value < 0.05 was considered significant.

## Results

Of the 111 patients operated for acute abdomen, 62 were males, 49 were females. Average age was 17.7 years (range 2–75), but more than half of the patients (54.9%) were less than 15 years old, and more than a third of these were less than 7 years old.

All of the patients had been brought to the emergency room for abdominal pain, and in 80% of the cases this was associated in previous days with diarrhoea and fever.

Blood count data for the day of laparotomy was available for only 70 patients. White blood cell count showed leukocytosis (WBC > 10000/mm^3^) in 28 cases (40%), normal white cell count (WBC = 3500-10000/mm^3^) in 35 cases (50%), and neutropenia (WBC < 3500/mm^3^) in 7 cases (10%). A haemoglobin level < 10 g/dl was seen in 44.2% of cases.

Of the 111 patients operated for suspected typhoid intestinal perforation, 104 presented peritonitis due to ileal perforation; in the remaining 7 cases there was only free peritoneal fluid with adenomesenteritis, with no evidence of perforation. Thus the clinical diagnosis of intestinal perforation was correct in 93.6% of cases. There were no large bowel perforations.

**Figure 3:**
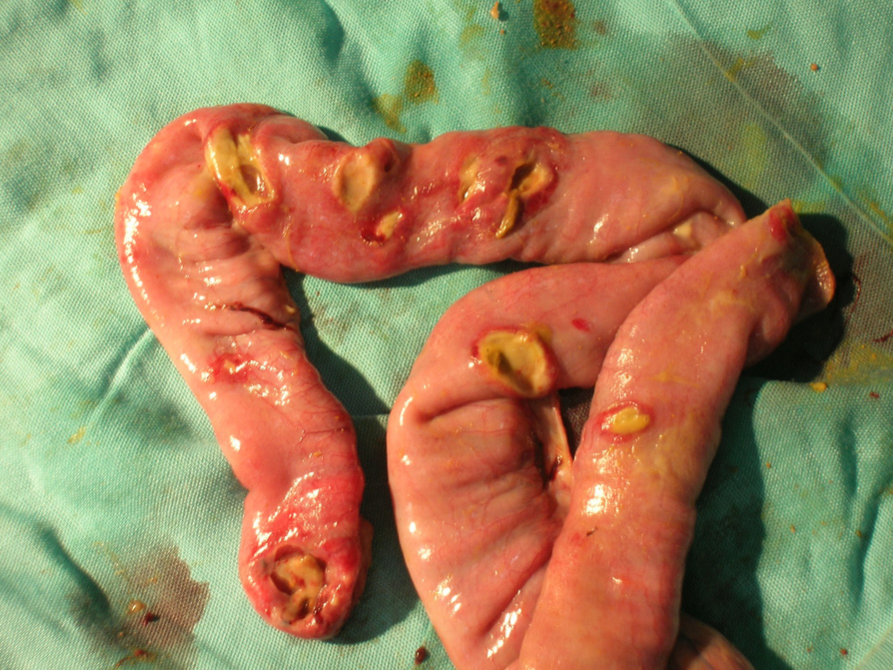
Multiple typhoid ileal perforations.

As regards the number of perforations, in the 104 perforated patients, at initial laparotomy there was a single perforation in 59.6%, two perforations in 23.0%, and more than 2 perforations in 17.3%. In 6.7% of cases there were 6 or more perforations (Figure 3). In the great majority of patients (82.6%) there were no more than 2 perforations.

The distance of the perforations from the ileocaecal valve was not specified for 19 of the 104 patients (18.2%). Of the 85 for whom the distance was recorded, in 37.6% the perforations were within 20 cm of the ileocaecal valve, in 51.7% between 20 and 30 cm, and in 10.5% more than 30 cm from the valve.

Pus was present in the abdomen for 57.6% of patients, and in 81.4% enteric content was found in the peritoneal cavity.

**Figure 4:**
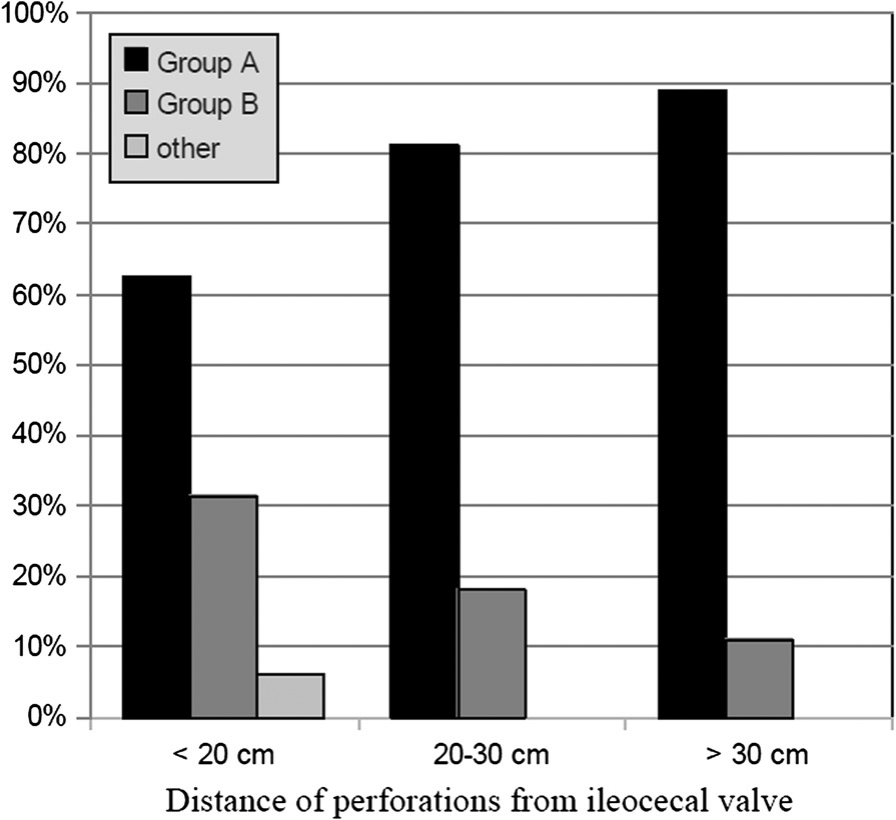
**Site of perforations and surgical technique adopted.** Group A: primary repair; Group B: intestinal resection with anastomosis; Other: other procedures.

Of the 111 patients who underwent surgery, 75 had primary repair (Group A) and 26 had ileal resection with anastomosis (Group B); 3 patients had other procedures (ileostomy on Foley catheter; primary repair associated with ileocaecal bypass; primary repair with proximal ileostomy); 7 patients had exploratory laparotomy only, because they had no perforations. Thus primary repair was the technique most frequently adopted in cases of perforation (72.1% of cases). In the 26 patients who underwent intestinal resection (Group B) an ileoileal anastomosis was created in 16 cases (61.5%), while ileocolic anastamoses were created in 10 cases (38.4%). Figure 4 relates perforation site to type of surgery adopted.

Although all but one of the resections were performed on patients with perforations ≤ 30 cm from the ileocaecal valve, we did not observe a significant correlation between site of perforation (> or < 30 cm ) and surgical procedure (primary repair or intestinal resection) (p = 0.35).

**Figure 5:**
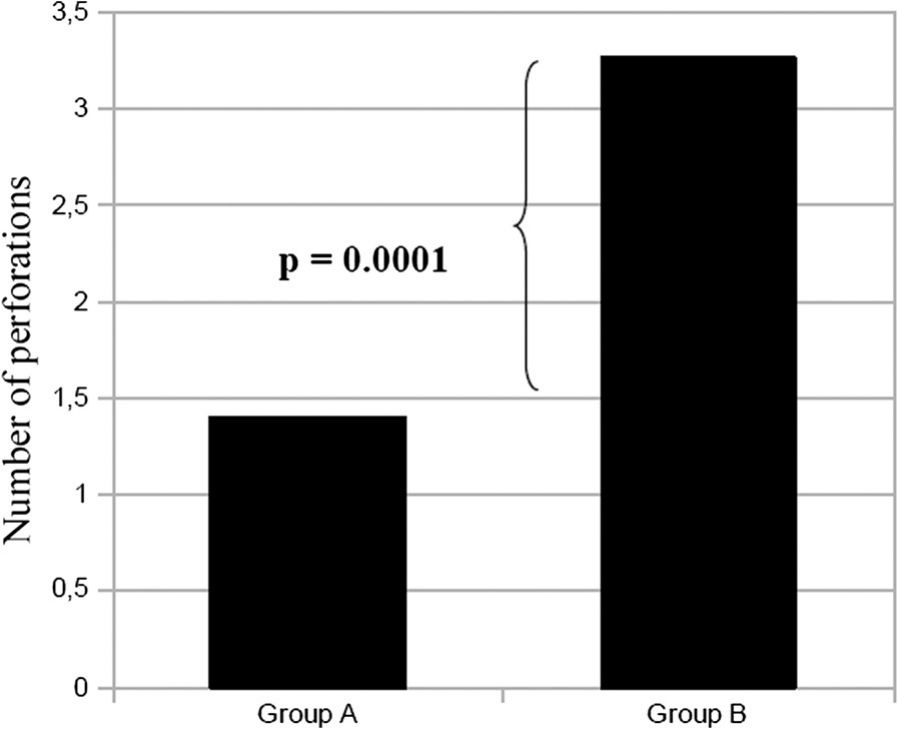
Number of perforations (average) in Group A and Group B at initial surgery.

However we did observe a correlation between surgical procedure and number of perforations. There were 85 perforations among the 26 Group B patients (average: 3.26), while there were 105 in the 75 Group A patients (average: 1.4) (p = 0.0001) (Figure 5), which is likely the reason for which the surgeon decided to perform an intestinal resection.

Thirty-seven of the 111 patients (20 from Group A, 10 from Group B and 7 other patients) underwent only the initial laparotomy, either because they died, were discharged, or were lost at follow-up after surgery. For the remaining 74 patients (54 in Group A, 16 in Group B and 4 other), laparostomy revision was carried out every 48–72 hours after the initial laparotomy.

The average number of laparostomy revisions was 3.47 (range 1–12). 37.8% of the patients had 3 or more revisions.

Laparostomy revisions showed complications in 8 Group A patients (8/54, 14.8%) and in 8 Group B patients (8/16, 50%) (p = 0.003). There were 6 new perforations and 2 primary suture dehiscences in group A patients; 3 new perforations and 5 dehiscences of the anastomosis made at first laparotomy were observed in group B. Six of these 16 patients presented recurrent dehiscence of primary suture or anastomosis at successive following laparostomy revisions (2 Group A patients and 4 Group B patients).

As regards treatment of complications, in the cases of primary repair dehiscence, re-suturing was carried out. In the cases of anastomotic dehiscence, re-suturing was carried out in only one case, while the other cases required a completely new anastomosis.

**Figure 6:**
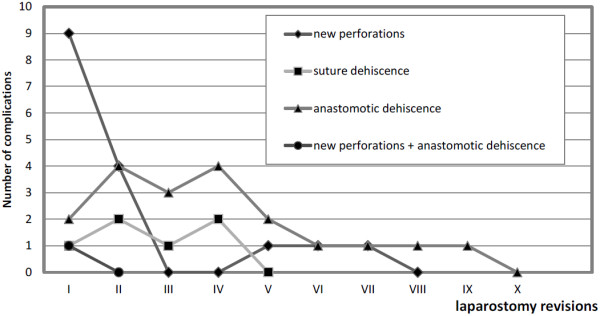
Complications observed at laparostomy revisions.

Figure 6 shows the distribution of complications observed at each laparostomy revision. From the table it can be seen that the number of new perforations decreases greatly after the third laparostomy revision, whereas the number of anastomotic dehiscences (frequently observed in the same patient) decrease only after the sixth revision.

In order to verify which, if any, clinical and laboratory parameters observed at initial laparotomy had an influence on the number of laparostomy revisions, we considered as potential risk factors: age; number of perforations; distance of the perforations from the ileocaecal valve, type of surgery; WBC count; haemoglobin level (Table 1).

**Table 1:** Correlation between number of laparostomy revisions and clinical parameters observed at first laparotomy (excluding patients who died or who were lost at follow-up after first laparotomy)

**Number of revisions**						**p**
	**1**	**2**	**3**	**4**	**>4**	
**Age** (average)	18.2	17.0	22.2	13	18	0.61
**Number of perforations at initial surgery** (average)	1.89	1.51	1.63	2	2.06	0.92
**Distance from the ileocaecal valve**						
< 30 cm (55 cases)	17	18	7	1	12	0.07
	(30.9%)	(32.7%)	(12.7%)	(1.8%)	(21.8%)	
20-30 cm (35 cases)	8	14	5	1	7	0.09
	(22.8%)	(40%)	(14.2%)	(2.8%)	(20%)	
> 30 cm (5 cases)	0	3	2	0	0	0.06
	–	(60%)	(40%)	–	–	
**Type of initial surgery**						
Primary repair (54 cases)	15	22	10	0	7	0.005
	(27.7%)	(40.7%)	(18.%)	–	(12.9%)	
Resection and anastomosis (16 cases)	3	3	1	1	8	0.023
	(18.7%)	(18.7%)	(6.2%)	(6.2%)	(50%)	
**WBC (average) at initial surgery**	7706	10500	10222	–	10750	0.58
**Hb (average) at initial surgery**	9.7	10.2	10.1	–	9.8	0.37

From an analysis of Table 1 it can be observed that age, number of perforations, haemoglobin concentration and WBC count at initial laparotomy do not correlate with the number of laparostomy revisions. Nor does perforation site correlate with the number of revisions, even though the patients who presented perforations more than 30 cm from the ileocaecal valve received at most 3 revisions, and 21.8% of patients with perforations less than 30 cm from the valve received more than 4 laparostomy revisions. However, resection with anastomosis (Group B) does correlate with a higher number of laparostomy revisions (p = 0.023), compared to the primary repair (Group A) (p = 0.005). In fact, 50% of patients who had resection with anastomosis (Group B) had more than 4 revisions, compared to only 12.9% of the primary repair patients (Group A). This confirms that, for patients with typhoid peritonitis, resection with anastomosis is characterised by greater morbidity than primary repair.

As regards mortality, it was possible to calculate this only for the 84 cases for which we had the final results, out of the total of 111 cases that underwent surgery. Table 2 shows the relationship between mortality and type of surgery, as well as certain clinical parameters. There was no statistical difference in terms of mortality between Groups A and B: 40% (8/20) vs 31% (18/58), p = 0.13. It should be noted that more than half of the patients who underwent only the initial laparotomy died. The greater mortality for these patients (p = 0.021) is most likely due to the fact that their general condition was so serious that they died before they could undergo any revision. In fact, in the patients who died after the initial laparotomy, 84.6% had pus in the peritoneal cavity, which is indicative of late treatment (advanced state of sepsis) and correlates with a significantly higher mortality (p = 0.0001). This datum could, however, be strongly influenced by the fact that the group of patients who underwent only the initial laparotomy also included the largest number of patients lost at follow-up.

**Table 2:** Mortality and various clinical parameters

**Clinical and laboratory parameters**	**Died (%)**	**p**
Type of surgery		
Primary Repair (Group A) *	31%	0.13
Intestinal resection + anastomosis (Group B)**	40%	
Total interventions (initial surgery plus laparostomy revisions) per patient (***)		
1 intervention	56.5%	0.021
2 interventions	29.4%	
3 interventions	15%	
4 interventions	20%	
> 4 interventions	21.4%	
**Age**		
Age < 10 years	26.4%	0.46
Age > 10 years	34%	
**Sex**		
Males	30.6%	0.67
Females	31.4%	
**Number of perforations at initial surgery**		
Perforations ≤ 2	30.5%	0.72
Perforations > 2	41.1%	
**Site of perforations**		
Perforation distance from valve < 30 cm	31.0%	0.17
Perforation distance from valve > 30 cm	57.1%	
**Presence of pus in peritoneum at initial surgery**		
Presence of pus in peritoneum	84.6%	0.0001
Absence of pus in peritoneum	15.3%	
**Blood cell count at initial surgery**		
WBC > 10.000	25%	
WBC < 3.000	100%	0.003
WBC 3.000 – 10.000	21.6%	
**Hb at initial surgery**		
Hb < 9 g/dl	19%	0.32
Hb > 9 g/dl	26%	

(*) Group A: 26 patients, 6 lost to follow-up: 20 evaluable.

(**) Group B: 75 patients, 17 lost to follow-up: 58 evaluable.

(***) 1 intervention = initial laparotomy only.

2 interventions = first laparotomy + 1 laparostomy revision.

3 interventions = first laparotomy + 2 laparostomy revisions.

4 interventions = first laparotomy + 3 laparostomy revisions.

> 4 interventions = first laparotomy + > 3 laparostomy revisions.

Overall mortality, excluding patients who died after the initial laparotomy, was 21.2%.

Mortality does not, on the other hand, seem to be related to the number of laparostomy revisions: there is no statistically significant difference, for example, in mortality between patients who had 2 revisions and patients who had more than 4 (29.4% vs 21.4%).

Sex and age did not seem to influence mortality, nor did the number of perforations at initial laparotomy. Mortality is strongly related to neutropenia (all of the neutropenic patients died: p = 0.003) but the presence or absence of leukocytosis was not seen to be related to mortality. Nor is there a correlation between mortality and anaemia at onset, probably due to the possibility of performing blood transfusions at the hospital centre in Tanguièta.

## Discussion

Interest in typhoid peritonitis is justified by its high incidence in developing countries, its high mortality, and its prevalence in children and young people [9,21-25]. In fact, in our experience approximately 30% of the patients were under 7 years old.

Most of our patients presented a state of severe malnutrition, although for this retrospective study we have no relevant data. Nor was the presence of leukocytosis seen to be a useful indicator of the immune response, as neutropenia was encountered in only 10% of cases; however, we did encounter microcytic anaemia (Hb < 10 g/dl) in 43.6% of the patients.

In spite of the scarcity of diagnostic tools, in our patients with acute peritonitic abdomen the indication for laparotomy was made correctly in 91.8% of cases, solely on the basis of clinical examination. Leukocytosis was not useful for diagnosis, being present in only 40% of these typhoid peritonitis patients. On the other hand, it was useful to spend adequate time to resuscitate and stabilise the patient before undertaking surgery, as suggested by others [3,15-17].

In such patients the number of typhoid perforations is widely variable (range 1–7) in literature [15,16,26] and in our experience we observed one case with 11 perforations (Figure 3). It is well known that the number and size of perforations have no relationship with the severity of symptoms [16], but it is nevertheless important to carry out a careful intestinal exploration at laparotomy [25]. In our experience, we found 2 or more perforations in 47.1% of our patients, and a number of these had more than 6. None of our cases showed signs of synchronous intestinal haemorrhage, which has been reported by others [16].

Regardless of the number of perforations, the finding of severe peritonitis is very frequent, and is often related to delayed access to the hospital (in our experience 81.4% of patients presented enteric content in the peritoneal cavity at first laparotomy).

Thus the first issue is to choose the surgical treatment because there are many factors to be considered. In fact, the choice of surgical treatment for ileal perforation remains controversial [15,16]. The types of surgical treatment recommended in literature include primary repair; simple excision of the edges of the perforation and closure; wedge resection and closure; segmental resection with primary end-to-end anastomosis; and right hemicolectomy with ileocolic or ileotransverse anastomosis [10,16]. In summary, we can say that there are two prevalent surgical procedures: primary repair and intestinal resection with anastomosis. Rahman and Atamanalp [10,21] found no correlation between the surgical procedures adopted and mortality. On the other hand, some others [27,28] found the rates of mortality and morbidity in resection-and-anastomosis patients lower than in primary repair patients. Beniwal has suggested primary repair as the first choice of treatment [7], as have others who reported a reduction in mortality [9,10,13,18,26,29-32]. Ileostomy might also be proposed among the options but we believe that it should be reserved for selected, very serious cases in which the macroscopic condition of the intestine, due both to typhoid disease and to peritonitis often neglected for hours or days, make any kind of repair impossible [10,25].

The choice between primary repair of the perforation (or perforations) and resection of the tract involved can be conditioned by various factors, some objective (macroscopic intestinal condition, number and site of perforations, severity of peritonitis, etc.) and others subjective, depending on the surgeon's experience.

In principle we can affirm that a single perforation should be sutured and the peritoneal cavity should be irrigated [11]. On the other hand, in a case of multiple perforations, close together, segmental resection with anastomosis is to be preferred [9,25].

As we have said, however, perforations can be highly variable in number, and they are often not close together. In our experience primary repair was the most widely used technique (72.1% of cases), and the choice was certainly influenced by the number of perforations if we consider that 72% of the primary repair patients had only one, with an average of 1.4 perforations in Group A and 3.26 in Group B (see Figure 5). However, primary repair was also adopted in cases of multiple perforations, which was the case for approximately 30% of our Group A patients. Primary repair is certainly the simplest and quickest technique, and can therefore in theory be performed by any surgeon.

Another factor believed by some to be relevant in the choice of surgical treatment is the distance of the perforation from the ileocaecal valve. In fact the valve can develop a condition of hypertension above it, that is, precisely in correspondence to the area perforated and repaired (by suture or resection and anastomosis), which could be considered a risk factor for dehiscence.

In our experience, almost 90% of perforations were sited within 30 cm of the ileocaecal valve, a situation confirmed in literature [33]. If we correlate the distance of the perforations from the ileocaecal valve with the type of surgery performed (see Figure 4), we see that primary repair was the most frequently adopted technique, regardless of the distance of the perforations from the valve, while resection with anastomosis was mostly performed for perforations less than 20 cm from the valve. However there was no statistical significance (p = 0.35).

The surgical choice probably depends on both parameters: number of perforations and site, taken together. In fact, 90% (9 out of 10) of patients with perforations less than 20 cm from the valve and subjected to resection had, in effect, multiple perforations, with an average of 2.8, while for the primary repair patients the perforations were multiple in only 25% of cases, and never more than 2 (average 1.35), regardless of the site. Thus, as it is logical to think, the tract of ileum < 20 cm from the valve, with multiple perforations – which corresponds to the most frequent site of typhoid perforations – was resected more often than repaired. However, there was no primary repair dehiscence, and only one dehiscence of an ileo-ileal anastomosis, at < 20 cm. Therefore the distance of the perforations from the valve seems to have little effect on the surgical choice and its results, while the presence of multiple perforations in this tract, close together, tends to influence the surgeon towards resection. This is confirmed by Mock’s study, in which the principle of adopting primary repair in all cases, in single or double plane, resulted in a mortality of 88% in cases of multiple perforations treated without resection [11].

As regards the strategy of creating a laparostomy and proceeding to revisions, we hypothesised its usefulness in malnourished patients with septic shock, for whom the risk of dehiscence and new perforations is very great, and the associated mortality is very high (67% according to Mock) [11]. It therefore seemed logical, in contrast with the opinion of some authors [13], to re-explore the abdomen rather than wait for clinical evidence of complications, evidence that can often be difficult to identify due to the scarcity of diagnostic tools and the consequent prolonging of a septic state.

In fact laparostomy revisions showed 16 patients with complications that were not yet clinically evident. There were 6 new perforations and 2 primary suture dehiscences in Group A patients (8/54, morbility 14.8%) while there were 3 new perforations and 5 anastomotic dehiscences in Group B (8/16, morbility 50%) (p = 0.003).

Contrary to what might be expected, resection does not seem to reduce the risk of new perforations. In our experience, the incidence of new perforations was more statistically significant in the Group B patients than in Group A (p = 0.01), but we have not found any data in literature concerning incidence of new perforations.

The incidence of new perforations decreases noticeably after the third revision, but the number of anastomotic dehiscences remains high (see Figure 6), although these often occur in the same patients; it can be supposed that the persistence of malnutrition, peritoneal phlogosis, and the septic state hinder the healing of the intestinal wall. In other words the resection patients (Group B) show greater morbidity than the primary repair patients, and therefore require a greater number of laparostomy revisions (see Table 1). In this connection, the risk of recurrency of anastomotic dehiscence is so high in the same patient as to lead us to create an ileostomy once the first dehiscence is encountered. Indeed, Meier, Adensukamni and Onen suggest an ileostomy in cases with multiple perforations and severe peritoneal contamination [14,20,34]. Atamanalp is of the same opinion, especially in cases of intestinal ischemia, inflammation, and edema, but nevertheless reports greater mortality in ileostomy patients, probably not due to the ileostomy itself but rather to the extreme severity of clinical conditions in these patients [10]. Unfortunately we could not consider ileostomy as a valid option for our patients, because in a rural area it is virtually impossible to find the necessary devices.

The high incidence of anastomotic dehiscence in our experience differs from that reported in literature, where it seems to be on average less than 10% [15,21]. This might be explained by the early identification of dehiscences through systematic laparostomy revision. Mortality, as we have mentioned before, was high (30-40%) for our patients, not statistically different between Groups A and B (see Table 2). We must consider, however, that more than half of the patients who died, died after the initial laparotomy, probably due to the severity of their general condition. Unfortunately, we did not find, retrospectively, sufficient data to quantify preoperative patient malnutrition and severity of sepsis. High mortality in the first 24 hours (30%) was also observed in the experience of Mock [11]. Thus, if we exclude these patients, overall mortality is noticeably lower (21.2%), according with others [12,25].

We confirm that mortality is not higher in patients with a greater number of perforations at initial laparotomy, as reported by some authors [7,10,21,35] but not confirmed by others [7,11,20,25]. We did not, however, observe increased mortality for male patients, although this is reported by others [10,13,14,18,20] and attributed perhaps to the fact that males spend more time than females in outdoor activities.

As regards perforation site, few studies in literature correlate this datum to mortality [16]. In our experience mortality was not related to perforation site. On the other hand factors that correlate with greater mortality, are neutropenia and severe peritoneal contamination (84.6% of the patients who presented pus in the peritoneum initially, died) as is confirmed by data in literature [9,11,14,18,20,25,29,34].

This last datum can be interpreted as a result of late access of the patient to a hospital, which many authors also consider to be a relevant factor for increased mortality [9,10,13,17,18,20,21,25],[34,36-38]. This delay is generally due to the distance from village to hospital, and by sociocultural factors (recourse to traditional medicine and healers).

Mortality is not, however, related to the number of operations performed (see Table 2). For example there is no significant difference in mortality between patients who had 2 revisions and patients who had more than 4 (29.4% vs 21.4%). Moreover, the surviving patients in both groups underwent, on average, a greater number of operations (Group A: 3.41, Group B: 4.83) than those who died (Group A: 1.81, Group B: 3.12) even though this is not statistically significant (p = 0.11 and 0.19, respectively). This could support our hypothesis that an apparently aggressive surgical strategy that provides for the adoption in principle of laparostomy with successive revisions may have, in these patients, a positive impact on survival.

Mortality is certainly strongly influenced also by the absence of intensive therapy (clinical monitoring, total parenteral nutrition, control of hydroelectrolytic balance, etc.), [15,18] which would surely reduce mortality in the immediate postoperative period. In fact, where postoperative recovery care is available, mortality is less than 5% [8,10,11,39].

## Conclusions

Typhoid perforations constitute an extremely serious clinical condition, especially for children. Primary repair is to be preferred, in principle, to resection with anastomosis, which is however indicated if the perforations are numerous and close together, regardless of the site. In our experience, patients who underwent resection with anastomosis showed greater morbidity, which is closely connected to anastomotic dehiscence, than primary repair patients. Thus, resection should be avoided whenever possible. Leaving the abdomen open (laparostomy) and carrying out successive revisions, makes it possible to identify rapidly not only any new perforations but also, and especially, anastomotic or primary repair dehiscences. In literature there are no other studies that have adopted this strategy. Although it may seem invasive to perform frequent operations in malnourished patients, the number of operations does not correlate with mortality; rather, the greater number of operations was seen in patients who had a favourable outcome.

## Consent

Written informed consent was obtained from the patient for the publication of this report and any accompanying images.

## Competing interests

There are no financial competing interests (political, personal, religious, ideological, academic, intellectual, commercial or any other) to declare in relation to this manuscript.

## Authors’ contributions

RC and GP designed the study. RC, AKB, DZ and GP performed surgical treatment. TH, RCG, CA and SA participated in postoperative care of patients, collection of data and review of literature. RC wrote the manuscript. All authors have and approved the final manuscript.

## Pre-publication history

The pre-publication history for this paper can be accessed here:

http://www.biomedcentral.com/1471-230X/13/102/prepub
